# Using Cluster Analysis to Explore Engagement and e-Attainment as Emergent Behavior in Electronic Mental Health

**DOI:** 10.2196/14728

**Published:** 2019-11-28

**Authors:** Samineh Sanatkar, Peter Andrew Baldwin, Kit Huckvale, Janine Clarke, Helen Christensen, Samuel Harvey, Judy Proudfoot

**Affiliations:** 1 Black Dog Institute School of Psychiatry University of New South Wales Sydney Australia

**Keywords:** eHealth, engagement, adherence, Web-based intervention, depression, anxiety

## Abstract

**Background:**

In most e-mental health (eMH) research to date, adherence is defined according to a trial protocol. However, adherence to a study protocol may not completely capture a key aspect of why participants engage with eMH tools, namely, to achieve personal mental health goals. As a consequence, trial attrition reported as non-adherence or dropout may reflect *e-attainment*, the discontinuation of eMH engagement after personal goals have been met. Clarifying engagement patterns, such as e-attainment, and how these align with mental health trajectories, may help optimize eMH design and implementation science.

**Objective:**

This study aimed to use clustering techniques to identify real-world engagement profiles in a community of eMH users and examine if such engagement profiles are associated with different mental health outcomes. The novelty of this approach was our attempt to identify actual user engagement behaviors, as opposed to employing engagement benchmarks derived from a trial protocol. The potential of this approach is to link naturalistic behaviors to beneficial mental health outcomes, which would be especially informative when designing eMH programs for the general public.

**Methods:**

Between May 2013 and June 2018, Australian adults (N=43,631) signed up to myCompass, a self-guided eMH program designed to help alleviate mild to moderate symptoms of depression, anxiety, and stress. Recorded usage data included number of logins, frequency of mood tracking, number of started and completed learning activities, and number of tracking reminders set. A subset of users (n=168) completed optional self-assessment mental health questionnaires (Patient Health Questionnaire-9 item, PHQ-9; Generalized Anxiety Disorder Questionnaire-7 item, GAD-7) at registration and at 28 and 56 days after sign-up. Another subset of users (n=861) completed the PHQ-9 and GAD-7 at registration and at 28 days.

**Results:**

Two-step cluster analyses revealed 3 distinct usage patterns across both subsamples: moderates, trackers, and super users, signifying differences both in the frequency of use as well as differences in preferences for program functionalities. For both subsamples, repeated measures analysis of variances showed significant decreases over time in PHQ-9 and GAD-7 scores. Time-by-cluster interactions, however, did not yield statistical significance in both subsamples, indicating that clusters did not predict symptom reduction over time. Interestingly, users who completed the self-assessment questionnaires twice had slightly but significantly lower depression and anxiety levels at sign-up compared with users who completed the questionnaires a third time at 56 days.

**Conclusions:**

Findings suggested that although users engaged with myCompass in different but measurable ways, those different usage patterns evoked equivalent mental health benefits. Furthermore, the randomized controlled trial paradigm may unintentionally limit the scope of eMH engagement research by mislabeling early mental health goal achievers as dropouts. More detailed and naturalistic approaches to study engagement with eMH technologies may improve program design and, ultimately, program effectiveness.

## Introduction

### Background

In 2005, Eysenbach [1] proposed a law of attrition to refer to the inevitability of substantial participant dropout (ie, dropout attrition) and discontinuation of program engagement (ie, nonusage attrition) from e-mental health (eMH) trials and called for rigorous examination of patterns of attrition to help clarify the impact, uptake, and dissemination of eMH programs. In reply, Christensen and Mackinnon [2] suggested that particular attention be given to users who derive symptom benefits from patterns of program engagement that deviate from trial protocols, particularly if nonusage attrition is common in randomized controlled trials (RCTs). Despite this, engagement in eMH research is still most frequently operationalized as adherence to an RCT protocol [3]. As such, much about program engagement outside the RCT context remains unknown. Where eMH programs are set up as a public health resource, understanding real-world engagement beyond protocol adherence is likely to strengthen eMH implementation science and help clarify patterns of eMH use in the general population. Findings of a recent systematic review of real-world engagement with eMH [4] suggest that attrition is also a common issue in naturalistic settings. Of the eleven interventions that were reviewed, 7% to 42% of sign-ups constituted moderate users who continually engaged with their eMH tool 4 to 6 weeks after registration. Only between 0.5% and 28.6% of users completed all modules or used the eMH program for more than 6 weeks after registration. As Price et al [5] pointed out, high dropout rates “limit the conclusions that can be drawn on the efficacy, feasibility, and public health impact of Web-based treatments.”

Inferring user adoption of open-access eMH programs from participant engagement in an RCT can be problematic. eMH program delivery in the RCT context likely involves more comprehensive and structured user support than would be sustainable in a community setting [6]. Indeed, when eMH program use is compared across RCT and open-access conditions, dropout attrition appears to be delayed or reduced in the RCT environment. For example, Christensen et al [7] provided a comparison between questionnaire completion rates of Australians who signed up to MoodGYM, an internet-delivered mental health intervention, either via the program website or as part of an RCT. The authors found that 66.5% of RCT participants completed a depression measure at registration and at least one depression assessment at follow-up, whereas only 15.6% of general public MoodGYM users did so. Such findings may be due, in part, to follow-up by researchers seeking to maximize study adherence rates [1]. For these reasons, Cavanagh [6] suggested that extrapolating adherence criteria from RCTs and applying these to open-access interventions may be problematic, as RCT protocols are at odds with the design and intent of such programs. More recently, Sieverink et al [8] proposed that treating all eMH programs as linear courses of treatment may create a black box of eMH, where mediating mechanisms and nonlinear patterns of engagement go unexamined, simply because they are off protocol. Yardley et al [9] suggested that researchers give attention to elucidating patterns of effective engagement—engagement that leads to the desired benefits—in addition to more standard RCT-defined adherence measures.

Recent efforts to define adherence and engagement have suggested avenues for improving eMH engagement research. Sieverink et al [10] reviewed definitions of adherence across over 60 eMH trials and found that studies providing clear adherence criteria (eg, completing 75% of modules) often provided no theory or data to support these criteria, and most studies defined adherence as simply *the more use, the better*. These authors recommended that future definitions of adherence include multiple metrics of engagement, be data-driven, and reflect both the goals of the technology (eg, symptom reduction) and the goals of the individual (eg, subjective states of well-being). In their recent scoping review, Pham et al [11] concluded that, although engagement metrics are increasingly consistent across studies, it is still unclear how patterns of engagement relate to mental health outcomes. Furthermore, Ryan et al [3] identified 8 theoretical frameworks in which adherence could be defined but found that few studies reported comprehensive program adherence data, with almost no consistency in the frameworks used to define engagement, attrition, and adherence. In line with Eysenbach [1] and Christensen and Mackinnon [2], Ryan et al [3] highlighted the ongoing risk of conflating engagement with adherence and the need to extend our understanding of eMH engagement beyond efficacy trials.

Eysenbach [1] proposed that a potential way forward was to examine the shape of attrition curves, a procedure that could hint at the presence of behavioral groupings, from low users to hardcore users. This suggests that one method for studying effective program engagement may be to identify *engagement profiles* within eMH cohorts and to examine whether program benefits (if any) vary between them. Some efforts have already been made to identify such usage profiles within RCTs. For instance, Donkin et al [12] examined which behaviors predicted clinically significant reductions in depressive symptoms among adherent users of a Web-based intervention targeting depression in cardiovascular disease. Activities per user session predicted significant improvement, whereas time spent on the intervention or the total number of modules and activities completed did not. High in-session engagers reported the best outcomes; however, medium and low in-session engagers experienced equivalent benefits. These results suggest that even within an adherent group the dose-response relationship is nonlinear and in-session engagement behavior may be more important to program efficacy than overall time-on-task. On the basis of their findings, Donkin et al [12] suggest that identifying when and for whom *treatment saturation* is achieved is an important goal for future eMH engagement research.

Techniques common in other areas of cognitive behavioral science may further help to identify eMH engagement profiles. Clustering and classification techniques can help identify emergent patterns of behavior [13]. Using engagement metrics that correspond to specific on-task behaviors, individuals can be grouped based on how they use their chosen eMH program. Once grouped, comparisons between these classes or clusters can be made to determine if different engagement profiles experience different benefits. The advantage of such an approach is that user groups emerge from the usage data. The results of such research are likely to better reflect user adoption behavior in an open-access environment with important implications for eMH implementation science.

### Objectives

The purpose of this study was to explore the utility of using clustering approaches to identify engagement profiles in eMH and to examine if such engagement profiles lead to different mental health outcomes. This approach has the potential to identify actual user engagement behaviors that may result in beneficial mental health outcomes and is likely to be especially informative when designing eMH programs for the general public. Cluster analytical approaches can help identify which short-term users, or dropouts, may be classified as e-attainers (ie, those who discontinue using an eMH program when ones’ personal mental health objectives have been attained, see [2]) and conversely, which sustained users may not achieve their desired mental health benefits. By understanding the link between usage behavior and mental health symptom progression, patterns of eMH tool cessation allow for a more differentiated view of attrition.

## Methods

### Target Program

*myCompass* is a self-guided Web-based mental health program available free to Australians [14]. It is designed for individuals experiencing mild to moderate symptoms of depression, anxiety, and stress and offers symptom tracking and interactive learning activities based on cognitive behavioral therapies. The “Daily Tracker” feature allows real-time tracking (by mobile phone or computer) of up to 3 areas of difficulty that may be emotional (eg, depression or anxiety), cognitive (eg, worry), or behavioral (eg, smoking) by measuring each on an 11-point scale ranging from low (0) to high (10). Users can also set to receive tracking reminders via SMS or email.

The myCompass learning activities aim to teach techniques for managing distress and improving well-being. Learning activities are delivered in 3 sessions and contain a session introduction, didactic content, interactive activities, and a practical home task. Each learning activity takes approximately 10 to 15 min to complete. myCompass also prompts users to complete an optional “self-assessment” questionnaire at 3 time points: the time of registration and at 28 and 56 days after sign-up. The self-assessment questionnaire (described below) measures symptoms of anxiety and depression and takes about 4 min to complete.

### Sample

The data were extracted from the usage data obtained from 43,631 adults who signed up to myCompass between May 2013 and June 2018. Upon signing up and as part of the Terms of Use agreement, all myCompass users consented to have their deidentified demographic and program usage information used for research purposes.

We conducted 2 sample extractions. In our first sample extraction, users were eligible for inclusion in analyses if they had completed self-assessment questionnaire data for all 3 time points (n=168). In our second sample extraction, we broadened our eligibility criteria to include individuals who completed the self-assessment questionnaire at the first 2 time points only (n=861 additional individuals). The resulting 1029 myCompass users included in this analysis had a mean age of 40.9 years (SD 13.45) and were mostly female (63.07%, 649/1029). Most resided in the Australian state of New South Wales (44.31%, 456/1029), followed by Victoria (19.63%, 202/1029) and Queensland (12.83%, 132/1029). Overall depression and anxiety scores, measured using the 9-item Patient Health Questionnaire (PHQ-9) [14] and the 7-item General Anxiety Disorder (GAD-7) Scale [15], were in the mild symptom ranges at sign-up (mean 10.68, SD 6.46 for depression and mean 8.58, SD 5.43 for anxiety).

### Engagement Metrics and Self-Assessment Questionnaire

#### Engagement Metrics

We used 5 usage metrics in our cluster analyses: number of user logins, number of daily trackers used, number of learning activities started, number of learning activities completed, and number of reminders received. All engagement data were extracted from and stored on secure myCompass servers, which record all user activity across the myCompass website. We took a conservative approach and defined login and tracking attempts that occurred in close succession (eg, 30 min apart for logins and less than 24 hours apart for daily tracking) as representing relogins because of system time-out. Hence, only the first instance of such records was included in our analyses.

#### Self-Assessment Questionnaire

The self-assessment questionnaire comprises the PHQ-9 and the GAD-7 and is used to measure symptoms of depression and generalized anxiety disorder, respectively. Both questionnaires ask individuals to rate symptom severity over the last 2 weeks on a 4-point Likert-type scale anchored *Not at all* (0) and *Nearly every day* (3). An example item for the PHQ-9 is “Little interest or pleasure in doing things,” and an example item for the GAD-7 is “Feeling nervous, anxious or on the edge.” Values on the PHQ-9 range from 0 to 27, and values on the GAD-7 range from 0 to 21. Both scales use cutoff scores of 5, 10, and 15 to identify individuals with mild, moderate, and moderately severe depressive and anxiety symptoms, respectively. The scales have been used extensively in clinical and research domains.

### Analysis Strategy

All analyses were computed in SPSS v.25 (IBM Corp). First, we employed a 2-step cluster analysis procedure to group users based on similarities in their myCompass engagement metrics. Cluster analysis is an exploratory classification technique that statistically identifies groupings in a dataset based on the degree of similarity between specified data points (for a more thorough discussion, see Jain et al [13]). A 2-step cluster analytical approach first constructs a cluster feature tree based on the similarity between data points and then uses a clustering algorithm to group the cluster feature tree nodes into an optimal number of clusters. This clustering procedure handles large datasets in a time-efficient manner and is quite robust against violations of assumptions.

As mentioned, the first cluster analysis contained only those 168 users who provided PHQ-9 and GAD-7 data at all 3 time points: sign-up, 28 days, and 56 days. To increase power and determine if our initial clusters would hold across a larger group, the second cluster analysis was broadened to contain users who provided mental health data at sign-up and at 28 days (but not at 56 days). In both analyses, we selected the loglikelihood distance measure and used the Bayesian information criterion as the clustering criterion. We let the program automatically compute the optimal number of clusters and used the default option of allowing no more than 15 clusters to be selected. To verify that the procedure had generated meaningfully different clusters, we used multivariate analysis of variance (ANOVA) to compare the clusters on all engagement metrics. We then employed repeated-measures ANOVA to examine if PHQ-9 and GAD-7 scores changed across time and differed between clusters.

## Results

### Analysis 1

#### Two-Step Cluster Analysis

The cluster analysis identified 3 distinct clusters (see Table 1). The first cluster, which we labeled “moderates,” comprised 74 users who, on average, logged into myCompass 11 times, used the daily tracker about 7 times, and completed 1 learning activity during 2 months of using myCompass. The second cluster, labeled “trackers,” contained 69 users who accessed myCompass about 36 times, used the daily tracker an average of 34 times, but showed comparable learning activity completion rates compared with the previous cluster across the 2-month time span. The third cluster “super users” represented 25 users who logged in approximately 39 times, used the daily tracker an average of 35.28 times, and showed markedly higher learning activity completion than the other 2 clusters (mean=5 modules completed). Multivariate ANOVA confirmed that the 3 cluster groups were significantly different on all usage variables (*P* values<.001), indicating that the cluster analysis identified distinct user groups.

**Table 1:** Results from multivariate analysis of variance looking at usage behaviors based on cluster membership of 168 myCompass users who completed the symptom screener at sign-up, 28 days, and 56 days.

Usage variables^a^ (count data)	Moderates (n=74), mean (SE)	Trackers (n=69), mean (SE)	Super users (n=25), mean (SE)	*F* test (*df*)	*P* value
Logins	11.00 (1.09)^b^	36.26 (1.13)^c^	38.92 (1.87)^c^	160.69 (2,165)	<.001
Tracking	6.91 (1.16)^b^	34.45 (1.21)^c^	35.28 (2.00)^c^	159.48 (2,165)	<.001
Modules completed	0.97 (0.13)^b^	0.80 (0.13)^b^	4.48 (0.22)^c^	116.41 (2,165)	<.001
Modules started	1.99 (0.15)^b^	1.33 (0.15)^c^	5.36 (0.26)^d^	93.10 (2,165)	<.001
Notifications received	5.53 (0.65)^b^	13.43 (0.67)^c^	9.80 (1.11)^d^	36.21 (2,165)	<.001

^a^Input variables are sorted by overall importance in this analysis, from highest to lowest.

^b,c,d^Differing superscripts indicate a significant difference at *P*<.05 for Bonferroni corrected multiple comparisons.

#### Repeated-Measures Analyses

**Figure 1:**
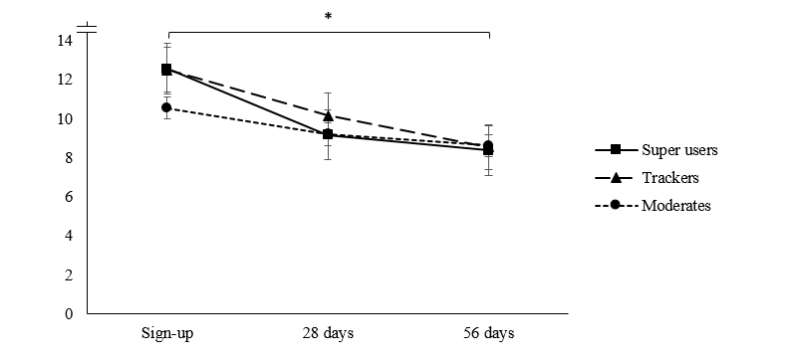
Mean trends of depression symptom progression, as measured by the Patient Health Questionnaire-9 item (range: 0-27), for myCompass usage groups at sign-up, 28 days, and 56 days.

**Figure 2:**
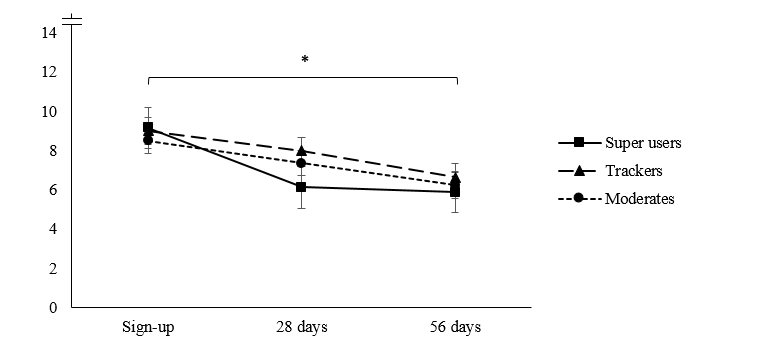
Mean trends of anxiety symptom progression, as measured by the Generalized Anxiety Disorder Scale-7 item (range: 0-21), for myCompass usage groups at sign-up, 28 days, and 56 days.

The Mauchly test of sphericity indicated that the variances of differences between groups were not equal. Hence, we proceeded to use Greenhouse-Geisser corrections to adjust the degrees of freedom. As shown in Table 2 and Figures 1 and 2, all clusters reported significant reductions in PHQ-9 and GAD-7 scores over time. However, the time-by-cluster interactions did not reach significance, indicating that symptom reduction over time did not vary by cluster in this initial sample.

**Table 2:** Repeated measures analysis of variances on Patient Health Questionnaire-9 item and Generalized Anxiety Disorder Scale-7 item scores at sign-up, 28 days, and 56 days compared across clusters (N=168).

Mental health variables		*F* test (*df*)	*P* value
**PHQ-9^a^**			
	Time	30.38 (1.87,309.23)	<.001
	Time × clusters	2.01 (3.75,309.23)	.10
**GAD-7^b^**			
	Time	23.84 (1.76,287.07)	<.001
	Time × clusters	1.05 (3.52,287.07)	.38

^a^PHQ-9: Patient Health Questionnaire-9 item.

^b^GAD-7: Generalized Anxiety Disorder Scale-7 item.

### Analysis 2

#### Two-Step Cluster Analysis

We repeated the steps of the first cluster analysis on the sample of 861 unique users who completed the PHQ-9 and GAD-7 at sign-up and at 28 days. As in the previous sample, the 2-step cluster analysis yielded 3 distinct user groups (see Table 3) that largely fit the moderates, trackers, and super user clusters previously identified. Multivariate ANOVA analysis confirmed that the newly derived 3 clusters also differed significantly on all usage variables (*P* values<.001).

#### Repeated-Measures Analyses

**Figure 3:**
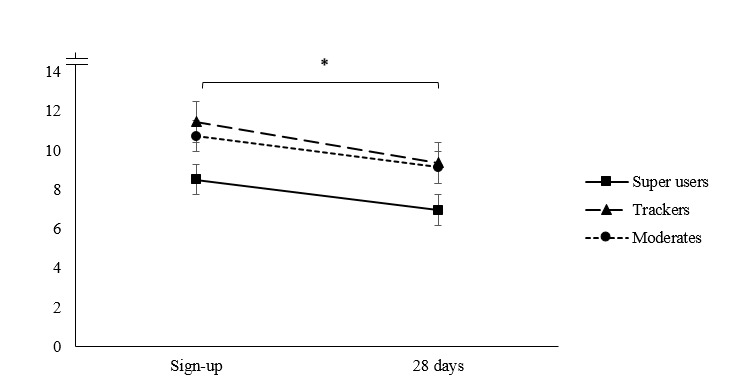
Mean trends of depression symptom progression, as measured by the Patient Health Questionnaire (range = 0 - 27), for myCompass usage groups at sign-up and after 28 days.

**Figure 4:**
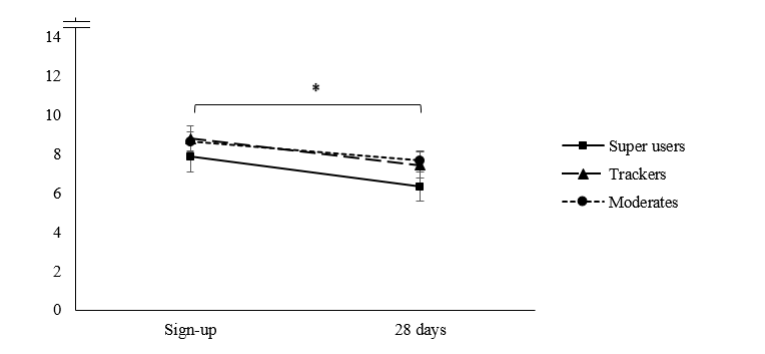
Mean trends of anxiety symptom progression, as measured by the Generalized Anxiety Disorder Scale-7 item (range: 0-21), for myCompass usage groups at sign-up and after 28 days.

Repeated measures ANOVAs showed significant decreases over time in PHQ-9 and GAD-7 scores; however, the time-by-cluster interactions were not significant (see Table 4 and Figures 3 and 4).

**Table 3:** Results from multivariate analysis of variance looking at usage behaviors based on cluster membership of 861 myCompass users who completed the symptom screener at sign-up and at 28 days.

Usage variables^a^ (count data)	Moderates (n=479), mean (SE)	Trackers (n=224), mean (SE)	Super users (n=158), mean (SE)	*F* test (*df*)	*P* value
Modules completed	0.18 (0.48)^b^	0.23 (0.40)^b^	2.08 (0.62)^c^	651.68 (2,858)	<.001
Logins	4.92 (0.33)^b^	19.02 (0.27)^c^	13.25 (0.43)^d^	960.89 (2,858)	<.001
Tracking	3.44 (0.34)^b^	17.96 (0.28)^c^	10.44 (0.44)^d^	930.79 (2,858)	<.001
Modules started	0.85 (0.07)^b^	0.77 (0.06)^b^	3.16 (0.09)^c^	443.56 (2,858)	<.001
Notifications received	5.22 (0.31)^b^	9.05 (0.25)^c^	6.28 (0.39)^d^	78.79 (2,858)	<.001

^a^Input variables are sorted by overall importance in this analysis, from highest to lowest.

^b,c,d^Differing superscripts indicate a significant difference at *P*<.05 for Bonferroni corrected multiple comparisons.

**Table 4:** Repeated measures analysis of variance on Patient Health Questionnaire-9 item and Generalized Anxiety Disorder Scale-7 item scores at sign-up and at 28 days compared across clusters.

Mental health variables.		*F* test (*df*)	*P* value
**PHQ-9^a^**			
	Time	66.99 (1,858)	<.001
	Time × clusters	0.67 (2,858)	.51
**GAD-7^b^**			
	Time	42.99 (1,858)	<.001
	Time × clusters	0.83 (2,858)	.44

^a^PHQ-9: Patient Health Questionnaire-9 item.

^b^GAD-7: Generalized Anxiety Disorder Scale-7 item, N=861.

#### Subsample Comparisons

In the second analysis, the super users reported the *lowest* PHQ-9 and GAD-7 scores at sign-up, contrasting with the symptom pattern observed in the first analysis. To determine if this effect was because of the inclusion of participants who completed self-report measures less frequently, we used ANOVA analyses to compare the 2 subsamples on their characteristics at the time of registration. Users who completed the self-assessment questionnaire 3 times were older, had slightly higher baseline PHQ-9 and GAD-7 scores, and used myCompass more in the first 28 days after signing up (see Table 5) compared with users who only completed the self-assessment questionnaire twice. Table 5 further shows differences between the characteristics of the 2 subsamples and the overall sample. Usage across the first 28 days after registration was significantly higher for myCompass users who revisited the platform over 1 or 2 consecutive months compared with the full sample that contained a large number of 1-time users (23,688/43,631, 54.29%).

**Table 5:** Means, SDs, and between-group statistics on demographic information and symptom severity at sign-up and usage behavior across 28 days.

Baseline characteristics and 28-day usage	Full sample (N=43,631), mean (SD)	2-month subsample (N=168), mean (SD)	1-month subsample (N=861), mean (SD)	*F* test	*P* value
Age	39.09^a^ (13.35)	41.29^b^ (14.14)	40.83^c^ (13.31)	9.37 (2,44657)	<.001
Females	1.69^a^ (0.47)	1.65^b^ (0.48)	1.63^b^ (0.49)	7.44 (2,44657)	.001
Baseline PHQ-9^d^	11.28^a^ (6.69)	11.64^a^ (6.53)	10.50^b^ (6.44)	5.99 (2,44657)	<.001
Baseline GAD-7^e^	8.98^a^ (5.73)	8.89^a^ (5.36)	8.52^b^ (5.45)	2.73 (2,44657)	.07
Logins	3.18^a^ (4.93)	14.64^b^ (8.82)	10.12^c^ (7.36)	1238.73 (2,44657)	<.001
Tracking	1.74^a^ (3.82)	12.75^b^ (9.49)	8.51^c^ (7.48)	1866.71 (2,44657)	<.001
Modules started	0.47^a^ (0.83)	1.64^b^ (1.39)	1.25^c^ (1.27)	517.99 (2,44657)	<.001
Modules completed	0.10^a^ (.47)	0.81^b^ (1.08)	0.54^c^ (.94)	518.18 (2,44657)	<.001
Notifications	3.19^a^ (3.33)	6.88^b^ (4.19)	6.41^b^ (4.11)	488.07 (2,44657)	<.001

^a,b,c^Differing superscripts indicate significant differences of *P*<.05 between groups.

^d^PHQ-9: Patient Health Questionnaire-9 item.

^e^GAD-7: Generalized Anxiety Disorder Scale-7 item.

## Discussion

### Principal Findings

The aim of this study was to investigate the feasibility and utility of using a clustering procedure to group eMH users based on their engagement behavior, and to determine if these engagement clusters reported any difference in mental health outcomes. Using a 2-step clustering procedure, we identified 3 clusters of engagement behavior, which emerged consistently across 2 subsamples in our community dataset. Although some patterns in the data suggest that increased overall engagement could be linked with more rapid symptom improvement, all users experienced equivalent symptomatic relief over the course of program use, irrespective of their pattern of engagement, which may speak to e-attainment.

The time-by-group differences were not statistically significant in analysis 1. As the study used naturalistic data that had already been collected—akin to archival data, we were unable to conduct an a priori power analysis to set a target sample size. Hence, it is possible that some of the statistical tests were underpowered and warrant replication in comparable but larger datasets. Therefore, any interpretations of the findings as they are presented in this study need to be made with caution. Bearing these considerations in mind, we would like to discuss interesting patterns that emerged in the data.

In analysis 1, “moderates” started out with slightly (though not significantly) lower depression scores at sign-up compared with “trackers” and “super users.” At 28 days, moderates and super users reported only mild depressive symptoms, whereas trackers remained just within the moderate symptom range. Only super users reported a clinically significant reduction in depressive symptoms (>3 points) at 28 days, though this likely reflects their higher PHQ-9 scores at the commencement of their program use. Notably, by 56 days all groups reported that their depressive symptoms had reduced into the mild symptom range.

Similar nonsignificant patterns emerged for anxiety symptoms in analysis 1. Moderates reported slightly lower anxiety levels at sign-up compared with trackers and super users. Super users showed the greatest reduction in anxiety scores at 28 days compared with trackers and moderates. At 56 days, all user groups had progressed further to the lower end of the mild anxiety range.

Consistent with pattern of results from analysis 1, super users in analysis 2 reported significantly lower depression scores at 28 days compared with moderates and trackers, though all groups reported depressive symptoms in the mild range by 28 days. Users’ anxiety symptom progression in analysis 2 also resembled the pattern of results for depression. At registration, average anxiety scores were in the mild anxiety range. At 28 days, all users progressed further toward the mid and bottom values of the mild anxiety category, with super users reporting significantly lower levels of anxiety than both moderates and trackers.

There were some interesting differences between our super users across the 2 analyses. In analysis 1 (across 2 months), super users appeared to report the *highest* distress at sign-up. However, in analysis 2 (across 1 month), super users appeared to report the *lowest* distress at sign-up compared with moderates and trackers. More generally, we found that the 1-month users from analysis 2 reported mild but significantly lower distress levels at sign-up compared with the more sustained 2-month users from analysis 1. It may be the case that some users experiencing greater distress at sign-up continued to use myCompass for longer than other users with milder symptoms.

Interestingly, belonging to a cluster in our study did not always seem to reflect symptom severity alone. Therefore, these engagement profiles may reflect both user need at sign-up and individual difference factors in technology engagement. For example, the super user engagement profile was identical across both analyses, but symptom severity at sign-up was different for 1-month super users relative to 2-month super users. This speaks to the complexity of eMH engagement behavior and underscores recommendations made by previous researchers [9,10] that individual user characteristics be included in conceptualizations of eMH engagement. If individuals ceased use of myCompass according to their symptom levels, we may have ended up with a less symptomatic group in our 1-month sample, relative to our 2-month sample. This systematic effect on program engagement would likely have resulted in symptom profile disparities between our analyses. Therefore, these seemingly contradictory results may speak to the phenomenon of e-attainment suggested by Christensen and Mackinnon [2], whereby users engage with an eMH tool as much and for as long as they need to reach their mental health goals. An alternative interpretation for the results would be that some of the standard components of eMH programs do not have the expected impact on symptom levels. For example, our results showed that trackers appeared to do as well as super users, which gives the impression that learning activities do not provide additional benefit beyond the effects of mood monitoring for some users. Given that substantial empirical evidence supports the cognitive behavioral therapies used in the learning activities, more data are required to better understand the unique benefits of specific eMH treatment components.

Our study provided an attempt at opening the black box of eMH [8] by moving beyond a priori definitions of adherence and, instead, inspecting behavior as it emerged from open-access program usage. Our findings suggested that, at least among our self-selected sample, individuals seem to engage with our eMH program in different but measurable ways that lead to equivalent mental health benefits. These findings align with a previous investigation conducted by Matthews et al [15]. Similar to the current investigation, the authors used cluster analysis to inspect naturalistic usage patterns among mobile phone users who downloaded and registered to a self-help app designed to help reduce anxiety symptoms. Findings indicated that all 4 usage groups identified experienced short-term decreases of anxiety symptoms irrespective of their engagement profile. Where mental health goals are achieved in a relatively short period of time, cessation of program use may create the impression of dropout across eMH websites and mobile phone apps.

Whereas the robustness of the RCT paradigm is crucial to efficacy science [16], it may unintentionally limit the scope of eMH engagement research in certain ways. For example, statistical practices that assume no benefit for participants who deviate from the trial protocol (eg, per-protocol or intention-to-treat with last-operation-carried forward analyses) may exclude individuals for whom program use is nonetheless beneficial. In addition, the prevailing RCT paradigms of symptom reduction or diagnostic remission may not reflect the usage goals or expectations of all eMH users. Although some eMH consumers seek treatment for a specific mental health condition, others may use eMH programs to navigate periods of general distress or to build resilience [17,18]. Many eMH users may be capable of determining when sufficient help has been accessed and subsequently discontinue without ever measuring symptoms or receiving a diagnosis [2]. However, within a traditional RCT framework, these so-called e-attainers are likely to be classified as nonadherent and therefore excluded from further analysis or aggregated with true nonusers in any subsequent per protocol analyses. Traditional RCT designs may be able to follow up participants who ceased using their prescribed eMH program and get an indication as to why participants opted out of the program. This approach was successfully implemented by Postel et al [19] who followed up RCT participants who prematurely ended their involvement with a Web-based intervention to reduce problem drinking. Interestingly, satisfaction with the treatment progress constituted the third most common reason for participants’ decision to drop out of the study, highlighting the potential importance of identifying whether noncontinuation is because of intervention success or failure. Although findings are preliminary, our results support Yardley et al’s [9] recommendations for identifying effective engagement and Pham et al’s [11] appeal to move beyond descriptive reporting of usage behaviors and examine the relationship between engagement and mental health outcomes. These efforts could provide a valuable supplement to traditional a priori definitions of eMH engagement.

### Limitations

Some limitations of our study must be acknowledged. First, the symptom change data were uncontrolled and therefore cannot be compared against a comparison condition as we inspected usage behavior in a naturalistic setting. Consequently, symptom reduction over time may be because of remission patterns rather than reflective of a treatment effect. Second, it is important to note that our clustering procedure is only one example of a categorization procedure, and other methods, such as latent class analysis, may yield different results. As mentioned above, discussing trends can be useful but further research is required to see if these effects can reach significance and are replicable in other datasets. The purpose of this study was not to establish best practice, rather it was to describe a novel way of inspecting usage behavior in the hope of encouraging similar approaches in the future that examine naturalistic behavior in addition to predetermined behavioral benchmarks. Finally, it is important to note that entry into the sample used for this study was dependent on users completing the symptom assessment scales at least twice. Therefore, there is at least one other group of users of eMH products not included in this analysis, those who do not engage at all following the initial assessment or who engage very little. The impact of this pattern of nonengagement remains unclear.

### Conclusions

The benefits of exploring real-world examples of engagement is to gain a more differentiated picture of how users navigate through the eMH space and, by doing so, to advance our understanding of how eMH tools might become more sophisticated and helpful companions in mental health. Learning from implicit engagement patterns can help inform and strengthen new computational techniques, such as machine learning, which aim to provide a personalized and situation-sensitive user experience in real time and are designed to motivate users to engage with their respective eMH tool until the desired mental health goals are reached and sustained.

Examination of real-world eMH engagement is required to assist differentiation between e-attainment and dropout and will have important implications for progressing the eMH space in a way that results in more widespread acceptance of eMH tools in the general public. Ultimately, a primary goal of eMH research is to remove obstacles to engagement for individuals who might otherwise benefit from eMH. A more nuanced view of how many variables such as limited access to the internet, a lack of fit between the needs and wants of users and the eMH program, and the achievement of individual goals (ie, e-attainment) will inform how future eMH technologies can better reflect the behaviors and desires of their users.

## Abbreviations

ANOVA: analysis of variance
eMH: e-mental health
GAD-7: generalized anxiety disorder questionnaire-7 item
PHQ-9: Patient Health Questionnaire-9 item
RCT: randomized controlled trial
